# Breeding decisions and output are correlated with both temperature and rainfall in an arid-region passerine, the sociable weaver

**DOI:** 10.1098/rsos.170835

**Published:** 2017-09-13

**Authors:** Rafael Mares, Claire Doutrelant, Matthieu Paquet, Claire N. Spottiswoode, Rita Covas

**Affiliations:** 1 CIBIO, Centro de Investigação em Biodiversidade e Recursos Genéticos, InBio, Laboratório Associado, University of Porto, Campus Agrário de Vairão, 4485-661 Vairão, Portugal; 2 INDICASAT-AIP, Ciudad del Saber, Panama City 0843-01103, Panama; 3 CEFE-CNRS, 1919 Route de Mende, 34293 Montpellier, France; 4 FitzPatrick Institute of African Ornithology, DST-NRF Centre of Excellence, University of Cape Town, Rondebosch 7701, South Africa; 5 Institute of Evolutionary Biology, School of Biological Sciences, University of Edinburgh, Edinburgh EH9 3FL, UK; 6 Department of Zoology, University of Cambridge, Cambridge CB2 3EJ, UK

**Keywords:** arid-zone ecology, breeding season length, breeding season temperature, onset of breeding

## Abstract

Animal reproductive cycles are commonly triggered by environmental cues of favourable breeding conditions. In arid environments, rainfall may be the most conspicuous cue, but the effects on reproduction of the high inter- and intra-annual variation in temperature remain poorly understood, despite being relevant to the current context of global warming. Here, we conducted a multiyear examination of the relationships between a suite of measures of temperature and rainfall, and the onset and length of the breeding season, the probability of breeding and reproductive output in an arid-region passerine, the sociable weaver (*Philetairus socius*). As expected, reproductive output increased with rainfall, yet specific relationships were conditional on the timing of rainfall: clutch production was correlated with rainfall throughout the season, whereas fledgling production was correlated with early summer rainfall. Moreover, we reveal novel correlations between aspects of breeding and temperature, indicative of earlier laying dates after warmer springs, and longer breeding seasons during cooler summers. These results have implications for understanding population trends under current climate change scenarios and call for more studies on the role of temperature in reproduction beyond those conducted on temperate-region species.

## 1. Introduction

Reproduction involves high energetic and nutritional demands associated with the production of eggs and the development of embryos and, in many species, with subsequent care of offspring. The ability to time reproduction to match the peak of resource availability using predictive environmental cues is thus expected to be under strong selective pressure [1–4]. For example, in northern, temperate regions, where seasons are well defined by cold winters and warm summers, studies on birds have shown that the onset of reproduction is mostly determined by temperature and day length [5–7]. Indeed, temperature is an important determinant of reproductive success for these species due to its effects on food availability, energetic demands and physiological state [5,8,9]. By contrast, in arid regions, rainfall can range widely within and between years and is expected to play a more important role in determining the incidence and timing of reproduction [10–12]. Few long-term studies, however, have investigated concurrently the influence of rainfall and temperature on breeding decisions and output in arid-region species [13,14].

In arid, seasonal environments, the onset of rains is typically correlated with a subsequent increase in food availability [15,16]. Many primary producers appear to fine-tune their investment in growth and flowering to the first rains [17,18], the timing of which varies greatly between years despite their seasonality [19]. Studies have thus shown strong correlations between the onset of rains and breeding, to the point where breeding is drastically reduced or even absent in years of extremely low rainfall [11,12,20,21]. Furthermore, rainfall over a given period may determine the length of the breeding season. For example, in birds, it appears to affect whether breeders are able to lay replacement clutches after failed nesting attempts and, in species that can lay multiple clutches within a single season, the number of successful breeding attempts [10,21,22].

Although patterns of temperature are more predictable than those of rainfall [19], the dramatic seasonal variation in temperature experienced by most tropical and subtropical arid regions may also have implications for reproduction. Minimum and maximum temperatures can also vary greatly from year to year, but whether and how this variation affects breeding decisions and output is poorly understood [20,21,23]. In temperate regions, where temperature is an important determinant of food availability and acts as a cue that triggers reproduction, warmer springs can lead to earlier laying dates [5,7]. By contrast, lower-than-average winter temperatures may affect female condition [24] and lead to fewer individuals breeding and to reduced output in the following breeding season [25,26]. In arid regions, temperature may also be used as a cue for breeding [23] and can exert a strong influence on food availability: lower-than-average temperatures can reduce primary productivity, independent of rainfall [27], and may directly affect insect activity patterns and phenology, for example by increasing developmental times [28]. Furthermore, recent studies in birds have shown that high temperatures can have negative impacts on foraging efficiency and condition, which appear to be independent of food availability [29–31]. Taken together, these studies suggest that temperature in arid regions is likely to play a greater role in the timing of breeding and in reproductive success than currently reported.

In this study, we use 9 years of breeding data from a wild population of sociable weavers (*Philetairus socius*) to investigate the effects of a suite of measures of rainfall and temperature on breeding decisions and output. Sociable weavers are facultative cooperative breeders that are endemic to the semiarid savannah regions of southern Africa [32,33], which are characterized by unpredictable rainfall and extreme temperatures [34]. Sociable weavers form large colonies of up to 500 individuals who share a massive communal nest with multiple separate chambers, occupied year-round by pairs or groups of up to seven individuals [32,33]. Chambers provide thermoregulatory benefits [35–37] and function as individual nests during breeding periods. Breeding occurs both year-round and seasonally, but its timing is likely to be determined by food availability, with experimental food supplementation leading to earlier laying dates and more individuals breeding [33,38,39]. As in other arid systems, reproductive output has been linked to seasonal rainfall [22], with some pairs laying up to nine clutches in a single breeding period [38]. However, it remains poorly understood whether the onset of breeding is, in fact, determined by rainfall, and whether and how both the amount and timing of rainfall influence other reproductive parameters. Furthermore, although low and very high temperatures are negatively correlated with adult survival in sociable weavers [40], the impact of temperature fluctuations on reproduction has yet to be determined in this system. Here, we examine the correlations of both rainfall and temperature, measured during winter, spring and throughout the breeding season, with the timing of onset and length of the breeding season, probability of breeding and reproductive output in sociable weavers.

## 2. Material and methods

### 2.1. Study site and population

The study was conducted using data from a wild population of sociable weavers at the Benfontein Nature Reserve (28°52′ S, 24°50′ E) in South Africa. The study area covers approximately 15 km^2^ of open, semiarid savannah and contains around 30 sociable weaver colonies, many of which have been regularly captured and ringed since 1993 [34]. The climate is characterized by low annual rainfall (432 ± 134 mm per year (mean ± s.d.), range 187–789 mm, as recorded from 1990 to 2013; South African Weather Service), the majority of which falls between October and April (over 35 mm per month, on average), and by widely ranging temperatures: 2–19°C (mean daily minimum and maximum, respectively; minimum recorded, −8.4°C) during the coldest months (June and July), and 17–32°C (maximum recorded, 40.9°C) during the hottest months (December to February). Breeding at the study site can start as early as August and extend to as late as June, but the majority of clutches (over 96% in this study) are laid between September and March.

### 2.2. Data collection

Our study focuses on six breeding seasons with complete data (1999, 2000 and 2010–2013) and includes three additional years (2002–2004) where data were only collected between August and December for the analysis of the onset of breeding (details below). Colonies were visited every 1–4 days, and the contents of individual chambers were inspected to obtain laying dates and the number of chicks that fledged (further details on the field methods are described in Covas *et al*. [22]). The nestling period lasts 21–24 days [38], but in order to avoid prompting premature fledging, chambers were inspected for the last time 17 days after the first egg hatched, and we refer to the survival of chicks up to this point as fledging [22]. Colony sizes were estimated during captures conducted before the start of each breeding season and here include adults aged 2 years or older, as yearlings rarely reproduce [34]. All encounters with snakes (the primary nest predators, principally Cape cobras, *Naja nivea*, and boomslangs, *Dispholidus typus* [22]) during visits to colonies were recorded to estimate colony-specific predation rates (for inclusion in the analyses of reproductive output, see details below). We excluded from our study all colonies in which food had been experimentally supplemented [33,39].

Daily rainfall and temperature data were obtained from the South African Weather Service. We used data from the Kimberley Airport weather station (28°48′ S, 24°45′ E; 11 km northwest of the study site) rather than locally collected data, as the latter were not available for every day of the study period. Where daily rainfall data were available for both the study site and Kimberley Airport (*n* = 479 days), they showed similar values, with a mean absolute difference and standard deviation of 1.7 ± 5.2 mm.

### 2.3. Statistical methods

The aim of our study was to determine the best climatic predictors of breeding decisions and output (namely, onset and length of the breeding season, probability of breeding, number of clutches laid and number of fledglings produced per season), among a suite of measures of rainfall and temperature (details below). Model selection was based on an information-theoretic approach, and for each analysis we constructed a candidate set of models (see the electronic supplementary material). We used Akaike's information criterion values [41] corrected for small sample sizes (AIC_c_) [42] and estimated AIC_c_ differences (Δ_AIC_, the model's AIC_c_ minus the minimum AIC_c_ across all candidate models). Models with Δ_AIC_ less than two were considered to be the ‘best’, opting for simpler models (those with fewer estimated parameters) when more than one model had Δ_AIC_ < 2 and similar fits to the data in terms of log-likelihood [42]. We used this stringent ΔAIC cut-off as we were attempting to find parsimonious models within candidate sets that included nested models and models where correlated climatic variables had been fitted separately into otherwise identical models (details below). Variance explained by our best models was estimated following the methods described by O'Quigley *et al*. [43] (proportion of randomness explained, *ρ*^2^) and by Nakagawa & Schielzeth [44] (total variance explained, *R*_(c)_^2^), for Cox proportional-hazards models and (generalized) linear mixed models ((G)LMM), respectively (descriptions of models are presented below). All statistical analyses were conducted in R v.3.2.1 [45], using packages coxme 2.2-5 [46] for Cox models and lme4 1.1-7 [47] for (G)LMMs.

Candidate models in each of the analyses detailed below were fitted with breeding season (year) and colony identity as random intercept terms to account for repeated measures. Pairwise correlations between potential predictors (fixed terms, detailed for each analysis below) were assessed using scatter plots and Pearson's correlation coefficients (*r*) [48], and terms with an absolute *r* greater than 0.5 were not included in the same model. Continuous variables fitted as fixed terms were standardized by subtracting the mean and dividing by 2 s.d., to allow the direct comparison of their corresponding estimated effect sizes, and to facilitate the interpretation of the effects of variables involved in interactions [49]. For all our best models, we present estimated effect sizes and their associated 95% confidence intervals (95% CI; calculated by subtracting and adding, from and to the effect size, 1.96 times its standard error, s.e.).

#### 2.3.1. Onset of the breeding season

We used laying dates from a total of 27 colonies (7–14 per season), monitored over nine breeding seasons, to investigate the effects of rainfall and temperature on time to laying of first clutches. We used mixed-effects Cox proportional-hazards models, which allowed us to assess the effects of fixed (e.g. winter temperature) and time-dependent covariates (e.g. daily temperature) on the probability that a clutch would be laid at a given time interval (i.e. day), while accounting for differences in the baseline hazard of each level of the random intercept terms incorporated in our models (see [6] for a similar analytical approach). Our analysis was restricted to the two-month period (28 August to 31 October) in which the earliest-recorded clutch and approximately 60% of first clutches were laid in each of our six complete breeding seasons (remaining first clutches were laid within the following seven months). As Cox models allow the inclusion of censored data, our analysis included all potential breeding pairs, regardless of whether they bred during the observation period. The number of potential breeding pairs within each colony was taken to be half the number of adults aged 2 years or older, assuming an equal sex ratio [50].

We first built two candidate sets of models for the onset of breeding to determine the best predictors among various measures of recent (a) rainfall (electronic supplementary material, table S1a) and (b) temperature (electronic supplementary material, table S1b). We then built a third candidate set of models (electronic supplementary material, table S1c) to test for additive effects of recent rainfall and temperature, using the best predictors obtained for each of the two climatic variables. In the rainfall candidate set (a), we included rainfall during 7, 15 and 30 days, with a lag of 6 days [51], before the focal day (mean (range) = 3 mm (0–60), 6 mm (0–69), 12 mm (0–77), respectively). In the temperature candidate set (b), we included: mean minimum winter (June to July) and early spring (August) temperatures (1°C (−1 to 4) and 3.3°C (1.1 to 5.3), respectively), minimum temperature on the focal day (8.8°C (−5.6 to 22.4)) and an adjusted minimum temperature on the focal day (average over the previous 30 days, estimated using linear weighting which gives smoothed daily measures of temperature more heavily influenced by recent temperatures; 7.6°C (0.9–13.9)). As rainfall and temperature are highly seasonal, our daily measures were strongly correlated with the time component in the models (day of the year). We therefore adjusted these time-dependent variables for day of the year before incorporating them into the models, by subtracting the day-specific mean values of rainfall or minimum temperature (estimated using data collected between 1990 and 2013). To account for the increasing variance in rainfall from August to October, we then divided the above adjusted daily measures of rainfall by day-specific standard deviations (see [52] for a similar method). We also controlled for colony size (31 adults (4–150)) in all our models, and input variables were allowed to interact with the time component when assumptions of proportional hazards were not met (e.g. for a categorical variable, the laying curves for each level should have similar shapes and proportional values over time [53]).

#### 2.3.2. Length of the breeding season

We used laying dates from a total of 24 colonies (7–14 per season), monitored over six complete breeding seasons, to assess the effects of rainfall and temperature on the length of the breeding season. For each colony, we estimated the length of the breeding season as the interval between the 10th and 90th percentile for all laying dates within a given season, rather than taking the first and last breeding attempt, in order to minimize the effect of outliers (see [54] for a similar method). The resulting number of days was fitted as the response variable in a candidate set of LMMs (electronic supplementary material, table S2).

Candidate models of the length of the breeding season and models in all subsequent analyses (detailed below) were fitted with one of the following climatic variables of interest: rainfall during the entire, typical breeding season (September to March; mean (range) = 374 mm (208–652)), rainfall in the ‘early breeding season’ (September to November; 58 mm (31–100)) or mean maximum temperature for the entire typical breeding season (30°C (29–31)). Mean minimum winter temperature (June to July; 1°C (−1 to 4)) was excluded as a predictor (here and from all subsequent analyses) as it had a near-perfect negative correlation with the above breeding season temperature (*r* = −0.89, *p* < 0.001) and was deemed to be of lesser importance due to its temporal separation from breeding. Rainfall in the previous breeding season (406 mm (226–652)) was also included as a predictor given its expected effect on current food availability [55]. We also controlled for colony size (35 adults (4–150)) and breeding start date (i.e. the 10th percentile for laying dates), and the interaction of colony size with the climatic variables fitted in the model.

#### 2.3.3. Probability of breeding

We assessed the effects of rainfall and temperature (as detailed in §2.3.2) on the number of pairs that bred per colony per season (data from 24 colonies across six breeding seasons). We considered a set of candidate GLMMs with binomial errors (electronic supplementary material, table S3) and fitted the number of pairs per colony that initiated a clutch (i.e. successes) and those that did not (i.e. failures) as the two-vector response variable, to account for differences in the number of potential breeders per colony (i.e. weighted regression [48]). Candidate models were also fitted with rainfall in the previous breeding season, the estimated length of a colony's breeding season and colony size (as detailed in §2.3.2), and the interaction of colony size with the climatic variable fitted in the model.

#### 2.3.4. Reproductive output

We assessed the effects of rainfall and temperature (as detailed in §2.3.2) on the total number of clutches laid and the total number of fledglings produced (i.e. chicks reaching 17 days old) per colony per season (data from 24 colonies across six breeding seasons). We considered sets of candidate GLMMs with Poisson errors (electronic supplementary material, tables S4 and S5) fitted with the predictor variables listed in §2.3.3 and with colony-specific predation rates to control for the effects of predation on reproductive success. Predation rates were estimated for each colony using data from nest failures attributable to snakes [22] and the method devised by Mayfield [56] for calculating nest mortality. Briefly, for each colony, the total number of nest predation events (i.e. predation of all eggs or chicks of a clutch or brood) was divided by the sum of nest days within a season (days elapsed between laying and nest failure or fledging for each clutch) to obtain a daily predation rate (mean (range) = 0.02 nests (0–0.08)).

## 3. Results

### 3.1. Onset of the breeding season

**Figure 1.**
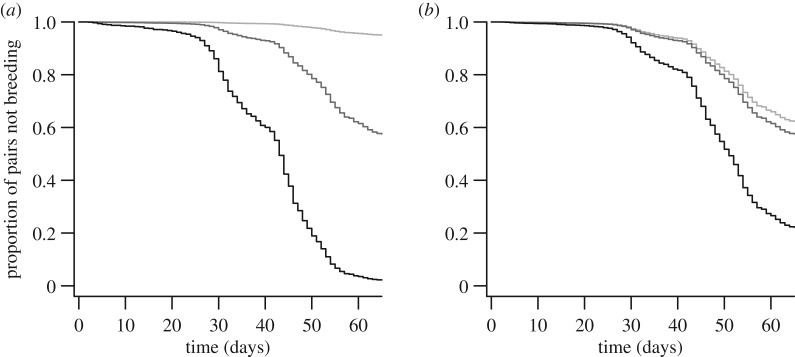
Survival-type curves showing time to laying of first clutches in relation to (*a*) mean minimum spring temperature and (*b*) recent rainfall measured over 7 days prior to each day within the observation period. Predicted curves were estimated from the model in table 1 (setting the predictor variables not plotted to their mean values) and correspond to temperatures and rainfall within the ranges recorded across the study years (light grey, dark grey and black lines for minimum, mean and maximum values, respectively). Day 0 corresponds to 28 August (earliest laying date in our dataset was 29 August).

Sociable weavers began breeding earlier when spring temperature and recent rainfall were higher (figure 1). The best model of the time to laying of first clutches (electronic supplementary material, table S1c, model 1: *Δ*_AIC_ = 0, *ρ*^2^ = 0.50) included mean minimum temperature in early spring and recent rainfall measured over 7 days prior to the focal day (table 1), as the best measures of temperature and rainfall, respectively, among those considered. Individuals in larger colonies were more likely to start breeding earlier in the season than those in smaller ones (table 1). The interactions between the time component and both spring temperature and colony size (table 1) indicated that their effects declined as the season progressed.

**Table 1.** Factors affecting time to laying of first clutches in sociable weavers. Results from the best Cox model in electronic supplementary material, table S1c (model 1), fitted with colony identity (variance = 0.26) and season (variance = 0.65) as random intercept terms.

fixed term	estimate ± s.e.	exp(Est.)	95% CI
spring temperature	4.49 ± 0.71	89.20	(22.1, 360.0)
spring temperature × time	−0.09 ± 0.01	0.92	(0.90, 0.94)
rainfall in previous 7 days	0.35 ± 0.09	1.42	(1.20, 1.68)
colony size^a^ (big)	1.38 ± 0.41	3.99	(1.78, 8.94)
colony size^a^ × time	−0.03 ± 0.01	0.97	(0.95, 0.98)

^a^Colony size fitted as a categorical variable (small < 24 individuals and big ≥ 24 individuals, based on the median colony size) to adjust for non-proportional hazards.

### 3.2. Length of the breeding season

**Figure 2.**
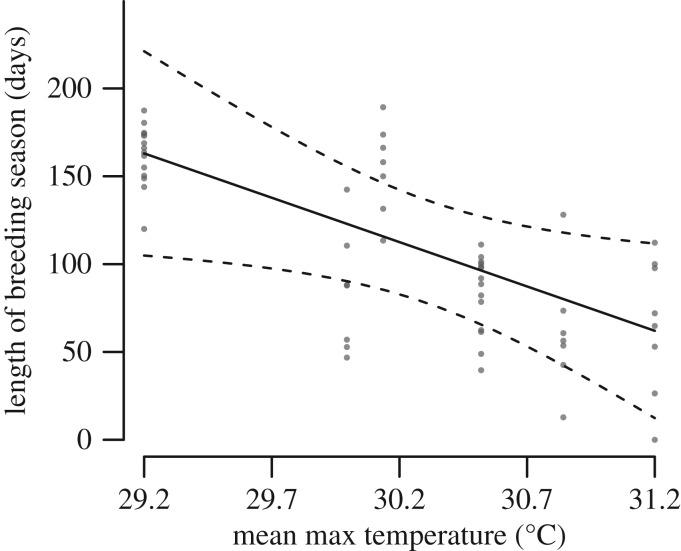
Length of the breeding season in relation to mean maximum breeding season temperature. Predicted values (solid line) and 95% CI (dashed lines) were estimated from the LMM in table 2, setting the predictor variables not plotted to their mean values. Points represent observed values for each colony, coloured light to dark according to the number of overlapping points.

Breeding seasons were longer when mean maximum breeding season temperatures were lower (figure 2 and table 2; electronic supplementary material, table S2, model 3: *Δ*_AIC_ = 0.48, *R*_(c)_^2^ = 0.88). Breeding seasons were also longer for larger colonies and when breeding started earlier in the year (table 2). Although alternative models that also included rainfall in the previous season and an interaction between temperature and colony size had marginally lower *Δ*_AIC_ values (electronic supplementary material, table S2, models 1 and 2), the difference in AIC_c_ with the simpler model (model 3) was less than 2, indicating little support for the inclusion of extra terms. The inclusion of early or total rainfall as predictors did not improve models over a null model without any climatic variables.

**Table 2.** Factors affecting the length of the breeding season, probability of breeding, and number of clutches and fledglings produced per colony in sociable weavers. Results from the best (G)LMMs in each candidate set (electronic supplementary material, tables S2–S5). Effect sizes and 95% CIs are presented for fixed terms (n/a, not applicable, when the term was not included in the candidate set of models), and variances for random intercept terms.

model components	length of breeding season	probability of breeding	number of clutches	number of fledglings
fixed term				
(intercept)	111.56 (82.03, 141.09)	−0.13 (−0.51, 0.25)	2.95 (2.70, 3.20)	1.48 (0.89, 2.07)
mean maximum temperature	−69.62 (−131.94, −7.31)			
total rainfall			0.43 (0.11, 0.75)	
early rainfall				1.62 (0.43, 2.82)
previous season rainfall		−0.93 (−1.52, −0.34)	−0.65 (−1.00, −0.31)	−2.28 (−3.44, −1.11)
colony size	15.21 (2.79, 27.63)	0.28 (−0.10, 0.66)	0.46 (0.28, 0.64)	0.43 (0.09, 0.76)
predation rate	n/a	n/a	0.34 (0.16, 0.52)	−0.71 (−1.09, −0.33)
breeding start date	−48.08 (−65.08, −31.09)	n/a	n/a	n/a
breeding season length	n/a		n/a	
random term				
colony ID	14.5	0.24	0.19	0.33
season	1271.6	0.08	0.03	0.35
error distribution	normal	binomial	Poisson	Poisson

### 3.3. Probability of breeding

**Figure 3.**
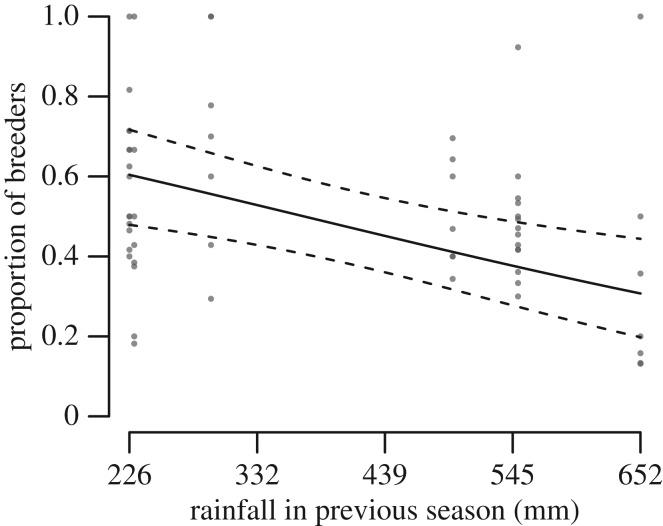
Probability of breeding in relation to rainfall in the previous breeding season. Predicted values (solid line) and 95% CI (dashed lines) were estimated from the GLMM in table 2, setting the predictor variable not plotted to its mean value. Points represent observed values for each colony, coloured light to dark according to the number of overlapping points.

The simplest model of the probability of breeding included rainfall in the previous season (electronic supplementary material, table S3a, model 9: *Δ*_AIC_ = 1.86, *R*_(c)_^2^ = 0.14). Fewer pairs bred following seasons during which rainfall was high (figure 3 and table 2). Alternative models that included an interaction between mean maximum breeding season temperature and colony size (model 6), or length of the breeding season and an interaction between total rainfall in the current breeding season and colony size (model 3), also had AIC_c_ values within 2 of the model with the lowest *Δ*_AIC_. However, these models had more parameters than the simpler model with only rainfall in the previous season.

A post hoc test, in which we tested for additional short-term effects of recent temperature and rainfall by incorporating them into the simplest model obtained above (electronic supplementary material, table S3a, model 9), indicated that rainfall in the 20 days before the peak of breeding activity was correlated with the probability of breeding (electronic supplementary material, table S3c: effect estimate (95% CI) = 0.53 (0.10, 0.96)). The models considered in the post hoc test also included the month during which the peak was recorded as a random intercept term, to account for the seasonal variation in temperature and rainfall (see details in electronic supplementary material, table S3b).

### 3.4. Reproductive output

**Figure 4.**
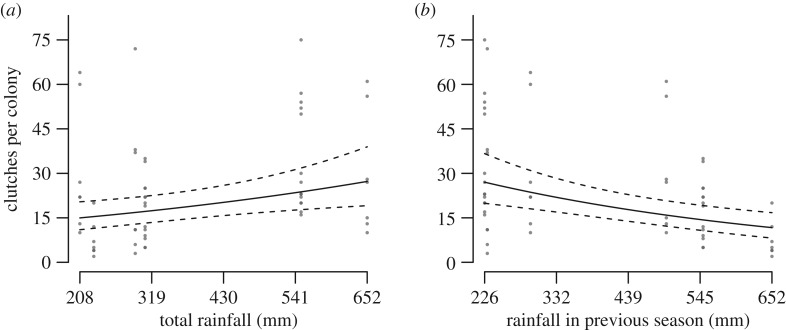
Number of clutches produced per colony in relation to (*a*) total rainfall in the current breeding season and (*b*) rainfall in the previous season. Predicted values (solid line) and 95% CI (dashed lines) were estimated from the GLMM in table 2, setting the predictor variables not plotted to their mean values. Points represent observed values for each colony, coloured light to dark according to the number of overlapping points.

The number of clutches produced per colony increased with rainfall over the current breeding season, but decreased after years of high rainfall (figure 4 and table 2 and simplest model in electronic supplementary material, table S4, model 2: *Δ*_AIC_ = 0.74, *R*_(c)_^2^ = 0.90). The number of clutches also increased with colony size and when predation was high. There was no effect of mean maximum temperature.

**Figure 5.**
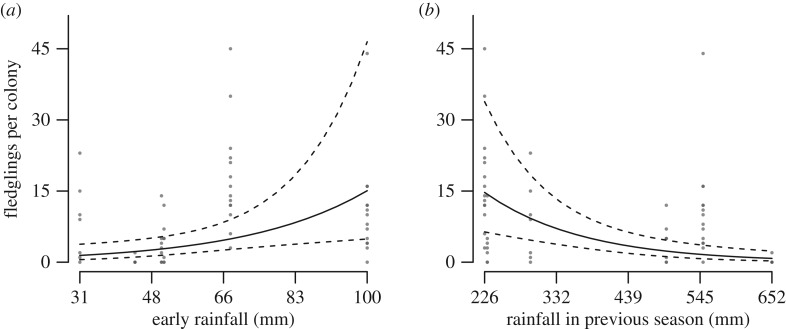
Number of fledglings produced per colony in relation to (*a*) early breeding season rainfall and (*b*) rainfall in the previous season. Predicted values (solid line) and 95% CI (dashed lines) were estimated from the GLMM in table 2, setting the predictor variables not plotted to their mean values. Points represent observed values for each colony, coloured light to dark according to the number of overlapping points.

More chicks fledged when rainfall was high early on in the breeding season, but fewer did so after years of high rainfall (figure 5 and table 2; electronic supplementary material, table S5, model 1: *Δ*_AIC_ = 0, *R*_(c)_^2^ = 0.87). The number of fledglings produced increased with colony size and was lower when predation was high. There was no effect of mean maximum temperature.

## 4. Discussion

Our results reveal an important role of temperature in the breeding phenology of arid-region birds: in sociable weavers, warmer springs were followed by earlier laying dates, and cooler summers were correlated with longer breeding seasons. Our results also confirm the importance of rainfall for breeding onset and success, but suggest that the timing of rainfall is as important as its quantity: for example, high rainfall early in the breeding season was associated with increased fledgling production, whereas rainfall across the entire breeding season was associated with more clutches being laid. Finally, our results suggest that rainfall may also have long-term effects: breeding probability and output were lower following years of high rainfall, independent of current rainfall. Overall, these results confirm the importance of rainfall for reproduction in arid-zone species. In addition, our findings on the effects of temperature on the breeding phenology of sociable weavers parallel those found in long-term studies on temperate-region passerines and highlight the importance of this often overlooked climatic variable on species living in arid environments.

In temperate regions, breeding patterns largely follow changes in day length, yet temperature plays a key role in determining the onset of breeding [5]. Long-term studies on blue tits (*Cyanistes caeruleus* [57]), great tits (*Parus major* [58]), long-tailed tits (*Aegithalos caudatus* [59]) and pied flycatchers (*Ficedula hypoleuca* [60]) suggest that birds use temperature as a predictive cue of food availability, with warmer springs typically leading to earlier breeding activity. In some cases, temperature appears to influence breeding decisions independently of other cues (e.g. leafing [61]), to the extent that mismatches between breeding activity and food abundance can occur in warmer-than-average years [1,7]. Our results align with those from previous studies, suggesting that temperature is also an important cue for birds breeding in arid regions [20,23], which may likewise be due to its effect on food availability. For example, the timing of swarming events in termites, one of the main prey species for arid-region passerines before the summer rains [62], may be influenced by spring temperatures [63,64]. Results from food supplementation experiments in sociable weavers [33,39] suggest that such an increase in food availability may ultimately determine the onset of breeding in arid-region passerines. While rainfall is also expected to precede an increase in food availability, seasonal changes in temperature are more predictable than the onset of rains, and may thus be a more reliable environmental cue that arid-region species can use to time their breeding activity.

Temperature can also affect the length of the breeding season. In temperate-region species, earlier start dates associated with warmer springs can result in longer breeding seasons [54]. In species that are able to produce multiple broods, these longer seasons may involve more clutches or longer intervals between clutches [54,65]. Our results for sociable weavers indicate that in addition to the correlation between earlier start dates and length of the breeding season, a reduction in mean summer temperature by 1°C was correlated with an extension of the reproductive window by up to 75%. Longer breeding seasons typically involved an increase in the mean number of clutches laid per colony, from eight clutches during the shortest breeding season to 35 during the longest. Note that this shift appears to be independent of colony size, as colonies were not necessarily larger at the start of longer breeding seasons. We found no such relationship, however, between length of the breeding season and interclutch interval at the individual level (range across pairs with two or more clutches, 17–39 days). The increase in clutches per colony could suggest that in sociable weavers, relatively cool summer temperatures increase reproductive output, as previously found in song sparrows (*Melospiza melodia* [66]), zebra finches (*Taeniopygia guttata* [67]) and lark buntings (*Calamospiza melanocorys* [68]). Indeed, in subtropical arid regions, high summer temperatures have been shown to reduce foraging efficiency and condition in southern pied babblers (*Turdoides bicolor* [29]) and southern fiscals (*Lanius collaris* [30]) and can ultimately suppress activities such as foraging, territorial behaviours and reproduction [69]. However, we found no clear correlations between temperature and number of clutches laid, fledglings produced or number of breeding pairs per colony. Thus, a simple, linear relationship between mean maximum temperature and a colony's reproductive output cannot be assumed based on the correlation between temperature and breeding season length. Given the likely negative impact of extreme temperature events on reproduction, and given that more broadly, the effects of temperature may be nonlinear [69,70] and inconsistent across species [68], further research into the role of temperature in determining reproductive output in this species is warranted.

As with temperature, the effects of rainfall on reproductive timing and output are likely largely due to its impact on food availability [71]. In arid environments, where water is a limiting factor for primary productivity and rainfall can be unpredictable, several studies have found a strong relationship between rainfall and reproduction [10,11,20,71]. Nevertheless, while the link between the amount of rainfall and breeding is well established, few studies have looked at the impact of the timing of rains across the breeding season. Using a proportional-hazards model which allows the incorporation of rainfall as a time-dependent variable (rather than fixed at an arbitrary point in time), we show that the probability of laying a first clutch in sociable weavers was correlated with rainfall during a moving, 7-day window. Similarly, we found that the probability of breeding at the peak of breeding activity was correlated with rainfall in the previous 20 days. Our results also suggest that the timing of rains affects total reproductive output, as rainfall early on in the breeding season was correlated with the number of fledglings produced over the entire season, but total rainfall was not. These results indicate that interannual variation in rainfall over long periods (e.g. during the entire breeding season) cannot alone explain the observed variation in breeding patterns. The importance of considering the effects of the timing of rainfall, in addition to its amount, on key life-history events is highlighted in recent studies on migration [72], breeding [12], growth [73] and survival [13,28,74] across multiple taxa.

Finally, our results showing that both breeding probability and reproductive output in sociable weaver colonies were lower after years of high rainfall, independent of current rainfall, suggest that rainfall may also have long-term effects. Given that reproductive output increases with current rainfall in sociable weavers, the decrease observed after favourable years could be due to a trade-off between current and future investment in breeding [75]. This is unlikely to be the case for all potential breeders in our study, however, as we included 2-year-old females which would probably not have bred as yearlings in the previous season [34] (but see [76]). Alternatively, lower reproductive output and breeding probability after years of high rainfall could be due to an increase in resource competition within or between colonies following favourable years [77,78]. Indeed, rainfall is correlated with adult survival in sociable weavers [40], and there is some evidence to suggest that the number of potential breeders (i.e. adults aged 2 years or older) drops below colony means after years of low rainfall (R. Mares *et al.* 2016, unpublished data). A longitudinal study of adult females in sociable weavers could shed further light on the potential long-term effects of rainfall on breeding decisions and output in this system.

Several recent studies have highlighted the effects that local changes in rainfall and temperature patterns due to global climate change are having on the timing of migration and breeding [3,79–82]. In arid regions, where rainfall and temperature range widely within and between years, the impact of climate change on survival and reproduction is expected to be exacerbated [29,83], yet there are few long-term studies on wild populations informing these predictions [13,14]. As a result, predictions on the fitness consequences that changes in rainfall and temperature patterns will have on arid-region species may not be robust [70,84]. Although the time span of our study is relatively short and our results should thus be treated with caution, our findings are largely consistent with those from a previous 17-year study that found that adult survival in the same sociable weaver population is negatively correlated with low rainfall and high temperatures [40]. Here, we show the importance of considering both rainfall and temperature for obtaining a broader view on how breeding patterns may be influenced by climatic variation. Our results suggest that the increase in temperature and reduction in rainfall for the central area of southern Africa, consistently predicted by different climate change models [85,86], are likely to lead to earlier laying dates, shorter breeding seasons and lower fledging success in sociable weavers. Shorter breeding seasons and lower fledgling success will clearly have a negative impact on reproductive output, but the effects of earlier laying dates may ultimately depend on the extent to which sociable weavers are able to maintain synchrony between the timing of breeding and increases in food availability in a rapidly changing environment.

## Supplementary Material

Candidate model sets

## Supplementary Material

Datasets

## Data accessibility

The datasets supporting this manuscript are available as the electronic supplementary material.
