# Supplementary material for: Breeding decisions and output are correlated with both temperature and rainfall in an arid-region passerine, the sociable weaver
Source: R Soc Open Sci. 2017 Sep 13;4(9):170835. doi: 10.1098/rsos.170835 (PMC5627122; doi:10.1098/rsos.170835)
Supplement: Candidate model sets [file rsos170835supp1.pdf]

Supplementary material

Candidate model sets

For all tables: Models ordered by AIC_c. Parameter estimates reported in the main article were obtained from the models highlighted in bold. log(*L*) = log-likelihood; *K* = number of estimated parameters; AIC_c = Akaike's Information Criterion adjusted for small sample sizes; Δ_{AIC} = the model's AIC_c minus the minimum AIC_c among candidate models.

Table S1a. Candidate models for best current rainfall predictor of time to laying of first clutches.

Model	Fixed terms	log(*L*)	*K*	AIC_c	Δ_{AIC}
1	R_7, C, C×*t*	-3963	5	7936	0.00
2	R_{15}, C, C×*t*	-3970	5	7950	14.12
3	C, C×*t*	-3978	4	7965	29.03
4	R_{30}, C, C×*t*	-3978	5	7966	30.19

Table S1b. Candidate models for best temperature predictor of time to laying of first clutches.

Model	Fixed terms	log(*L*)	*K*	AIC_c	Δ_{AIC}
1	T_s, T_s×*t*, C, C×*t*	-3929	6	7871	0.00
2	T_a, C, C×*t*	-3975	5	7959	88.63
3	C, C×*t*	-3978	4	7965	94.09
4	T_w, C, C×*t*	-3978	5	7965	94.31
5	T_d, C, C×*t*	-3978	5	7967	96.10

Table S1c. Candidate models for time to laying of first clutches including measures of rainfall and temperature.

Model	Fixed terms	log(*L*)	*K*	AIC_c	Δ_{AIC}
**1**	**T_s, T_s×*t*, R_7, C, C×*t***	**-3921**	**7**	**7857**	**0.00**
2	T_s, T_s×*t*, C, C×*t*	-3929	6	7871	14.03
3	R_7, C, C×*t*	-3963	5	7936	79.09

Tables S1a-c: Cox models of time to laying (*n* = 588 clutches laid within the observation period, August – October) fitted with season and colony identity as random intercept terms. For all models: C = colony size; R = rainfall over the previous 7, 15 and 30 days; T = minimum temperature: winter mean (w), spring mean (s), daily (d); moving average over the previous 30 days (a); *t* = time interval (i.e. days since 28 August). Measures of rainfall, and minimum daily and moving average temperatures were adjusted for day of the year and fitted as time-dependent covariates. Note: Although rainfall in the previous season is listed as a predictor in subsequent analyses, it was not included here because it violated the assumption of proportional hazards, even after allowing it to interact with the time component of the model.

Table S2. Candidate models for the length of the breeding season.

Model	Fixed terms	log(*L*)	*K*	AIC*c*	Δ_{AIC}
1	S, C, T, R_p	-262	8	543	0.00
2	S, C×T, R_p	-261	9	543	0.05
**3**	**S, C, T**	**-264**	**7**	**543**	**0.48**
4	S, C×T	-262	8	544	0.72
5	S, C×R_p, T	-261	9	544	0.98
6	S, C×R_t, R_p	-261	9	545	1.85
7	S, C, R_p	-264	7	545	2.12
8	S, C×R_t	-263	8	545	2.13
9	S, C	-266	6	546	2.61
10	S, C×R_p	-264	8	546	3.11
11	S, C, R_t, R_p	-264	8	547	4.49
12	S, C, R_t	-266	7	548	4.81
13	S, C×R_p, R_t	-263	9	548	5.53
14	C, R_e, R_p	-277	7	570	26.84
15	C, R_e	-278	6	570	27.11
16	C×R_e, R_p	-277	8	572	29.47
17	C×R_p, R_e	-277	8	572	29.53
18	C×R_e	-278	7	573	29.62

LMMs of length of the breeding season (n = 57 observations) fitted with season and colony identity as random intercept terms. For all models: C = colony size; S = breeding season start date; R = rainfall: total (t), early breeding season (e) and previous season (p); T = mean maximum breeding season temperature.

Table S3a. Candidate models for the probability of breeding.

Model	Fixed terms	log(*L*)	*K*	AIC*c*	Δ_{AIC}
1	C×R_p	-132	6	277	0.00
2	C×T, R_p	-130	7	277	0.07
3	L, C×R_t	-131	7	277	0.60
4	C×R_p, T	-131	7	277	0.72
5	C, T, R_p	-132	6	278	1.11
6	C×T	-132	6	278	1.33
7	C×R_p, R_t	-131	7	278	1.62
8	C×R_p, R_e	-131	7	278	1.76
**9**	**C, R_p**	**-134**	**5**	**279**	**1.86**
10	C×R_t, R_p	-131	7	279	2.13
11	C, R_t, R_p	-133	6	279	2.69
12	C, R_e, R_p	-133	6	280	3.35
13	C, T	-135	5	280	3.72
14	L, C	-135	5	282	4.89
15	C×R_e, R_p	-133	7	282	5.37
16	L, C, R_t	-134	6	282	5.50
17	L, C, R_e	-135	6	284	7.28
18	L, C×R_e	-135	7	286	9.19

GLMMs with binomial errors of the probability of breeding (*n* = 57 observations), fitted with season and colony identity as random intercept terms. For all models: C = colony size; L = length of the breeding season; R = rainfall: total (t), early breeding season (e) and previous season (p); T = mean maximum breeding season temperature.

Table S3b. Candidate models for the probability of breeding including additional effects of current climatic variables (post-hoc test).

Model	Fixed terms	log(*L*)	*K*	AIC*c*	Δ_{AIC}
**1**	**C, R_p, R_{20}**	**-125**	**7**	**267**	**0.00**
2	C, R_p, R_{40}	-126	7	268	1.15
3	C, R_p, R_{60}	-126	7	268	1.64
4	C, R_p, T_{30}	-126	7	269	2.43
5	C, R_p	-128	6	269	2.52

GLMMs with binomial errors of the probability of breeding (*n* = 57 observations), fitted with season, colony identity and month at which the peak of breeding activity (i.e. maximum number of breeders) was recorded as random intercept terms. For all models: C = colony size; R = rainfall: in the previous season (p), and 20, 40 and 60 days prior to the peak of breeding activity; T = mean maximum temperature over the 30 days prior to the peak of breeding activity.

Table S3c. Factors affecting the probability of breeding including current climatic variables (post-hoc test).

Fixed term	Estimate	(95% CI)
(Intercept)	-0.08	(-0.50, 0.34)
Colony size	0.23	(-0.25, 0.71)
Rainfall in previous season	-0.76	(-1.17, -0.36)
Rainfall in past 20 days	0.53	(0.10, 0.96)

Results from the best GLMM in Table S3b (model 1), fitted with colony identity (variance = 0.28), month (variance = 0.08) and season (variance = 0.00) as random intercept terms.

Table S4. Candidate models for the number of clutches laid per colony.

Model	Fixed terms	log(*L*)	*K*	AIC*c*	Δ_{AIC}
1	P, C×R_p, R_t	-230	8	480	0.00
**2**	**P, C, R_t, R_p**	**-232**	**7**	**480**	**0.74**
3	P, C×R_p	-232	7	481	1.36
4	P, C×R_p, T	-231	8	482	2.14
5	P, C×R_t, R_p	-231	8	482	2.27
6	P, C, R_p	-234	6	483	2.93
7	P, C, T, R_p	-233	7	483	2.94
8	P, C×R_p, R_e	-232	8	483	3.34
9	P, C, R_e, R_p	-234	7	484	4.67
10	P, C×T, R_p	-233	8	484	4.74
11	P, C, T	-236	6	485	5.33
12	P, C	-237	5	485	5.60
13	P, C, R_t	-236	6	485	5.71
14	P, C×R_e, R_p	-234	8	486	6.41
15	P, C×R_t	-235	7	487	7.20
16	P, C×T	-235	7	487	7.27
17	P, C, R_e	-237	6	488	8.08
18	P, C×R_e	-237	7	490	10.14

GLMMs with Poisson errors of the number of clutches laid (*n* = 56 observations), fitted with season and colony identity as random intercept terms. For all models: C = colony size; P = predation rate; R = rainfall: total (t), early breeding season (e) and previous season (p); T = mean maximum breeding season temperature.

Table S5. Candidate models for the number of fledglings produced per colony.

Model	Fixed terms	log(*L*)	*K*	AIC_c	Δ_{AIC}
**1**	**P, C, R_e, R_p**	**-170**	**7**	**357**	**0.00**
2	P, C×R_p, R_e	-169	8	357	0.33
3	P, C×R_e, R_p	-170	8	359	2.29
4	P, C, R_p	-173	6	359	2.53
5	P, C×R_p	-172	7	360	2.93
6	P, C, T, R_p	-172	7	360	3.47
7	P, C×R_p, T	-171	8	360	3.73
8	P, C×T, R_p	-171	8	361	4.38
9	P, C, T	-174	6	361	4.57
10	P, C, R_t, R_p	-173	7	361	4.79
11	P, C×R_p, R_t	-171	8	362	5.20
12	P, C×T	-173	7	362	5.46
13	P, L, C	-175	6	363	6.55
14	P, C×R_t, R_p	-172	8	364	7.13
15	P, L, C, R_e	-174	7	365	8.34
16	P, L, C, R_t	-175	7	365	8.75
17	P, L, C×R_e	-174	8	367	10.59
18	P, L, C×R_t	-174	8	368	11.11

GLMMs with Poisson errors of the number of fledglings (n = 56), fitted with season and colony identity as random intercept terms. For all models: C = colony size; L = length of the breeding season; P = predation rate; R = rainfall: total (t), early breeding season (e) and previous season (p); T = mean maximum breeding season temperature.
